# Atherosclerotic Components in Thrombi Retrieved by Thrombectomy for Internal Carotid Artery Occlusion Due to Large Artery Atherosclerosis: A Case Report

**DOI:** 10.3389/fneur.2021.670610

**Published:** 2021-05-28

**Authors:** Futoshi Eto, Junpei Koge, Kanta Tanaka, Takeshi Yoshimoto, Masayuki Shiozawa, Kinta Hatakeyama, Kazunori Toyoda, Masatoshi Koga

**Affiliations:** ^1^Department of Cerebrovascular Medicine, National Cerebral and Cardiovascular Center, Suita, Japan; ^2^Department of Neurology, National Cerebral and Cardiovascular Center, Suita, Japan; ^3^Division of Stroke Care Unit, National Cerebral and Cardiovascular Center, Suita, Japan; ^4^Department of Pathology, National Cerebral and Cardiovascular Center, Suita, Japan

**Keywords:** mechanical thrombectomy, cholesterol clefts, histopathology, thrombus, large artery atherosclerosis, acute ischemic stroke

## Abstract

**Introduction:** The correlation between the composition of thrombi retrieved by mechanical thrombectomy (MT) and stroke etiology is inconclusive. We describe a case with atherosclerotic components in thrombi retrieved by MT for acute internal carotid artery (ICA) occlusion.

**Case Presentation:** A 69-year-old man with acute onset of global aphasia and right hemiplegia was transferred to our institute. His baseline National Institutes of Health Stroke Scale score was 24. Magnetic resonance imaging demonstrated acute ischemic stroke in the left parietal lobe. Magnetic resonance angiography revealed occlusion of the left ICA. MT was attempted for acute left ICA occlusion. The initial angiography showed occlusion of the proximal ICA, while intraprocedural angiography revealed a large thrombus that extended from the cervical ICA to the intracranial ICA. Successful reperfusion was achieved by five passes using stent retrievers and an aspiration catheter. A large volume of red thrombus was retrieved by each pass. The final angiogram showed successful reperfusion with modified Thrombolysis in Cerebral Ischemia grade 2b and severe stenosis in the proximal ICA. Neck magnetic resonance imaging showed severe left ICA stenosis with a vulnerable plaque. Hence, his stroke etiology was determined as large artery atherosclerosis. Histopathological examination of the retrieved thrombi revealed atheromatous components, including cholesterol clefts, foam cells, and a necrotic core.

**Conclusions:** Atherosclerotic components in retrieved thrombi might provide useful clues for diagnosing stroke pathogenesis. Further studies are warranted to clarify the utility of assessing atheromatous components in retrieved thrombi in diagnosing stroke etiology.

## Introduction

Significant advances in the technology of mechanical thrombectomy (MT) have made it possible to perform analysis of the retrieved thrombus in acute ischemic stroke patients with large vessel occlusion (1). Although previous studies have reported the correlation between structural components of retrieved thrombi on histopathology (e.g., red blood cells, fibrin/platelet compositions) and stroke pathogenesis (1, 2), histopathological findings directly linked to the diagnosis of stroke etiology remain to be elucidated. Herein, we describe a case in which atherosclerotic components were found in thrombi retrieved by MT performed for acute internal carotid artery (ICA) occlusion. The histopathological characteristics of the thrombi seen in the present case might be key findings to diagnosing stroke due to large artery atherosclerosis (LAA).

## Case Presentation

### History and Clinical Examination

**Figure 1:**
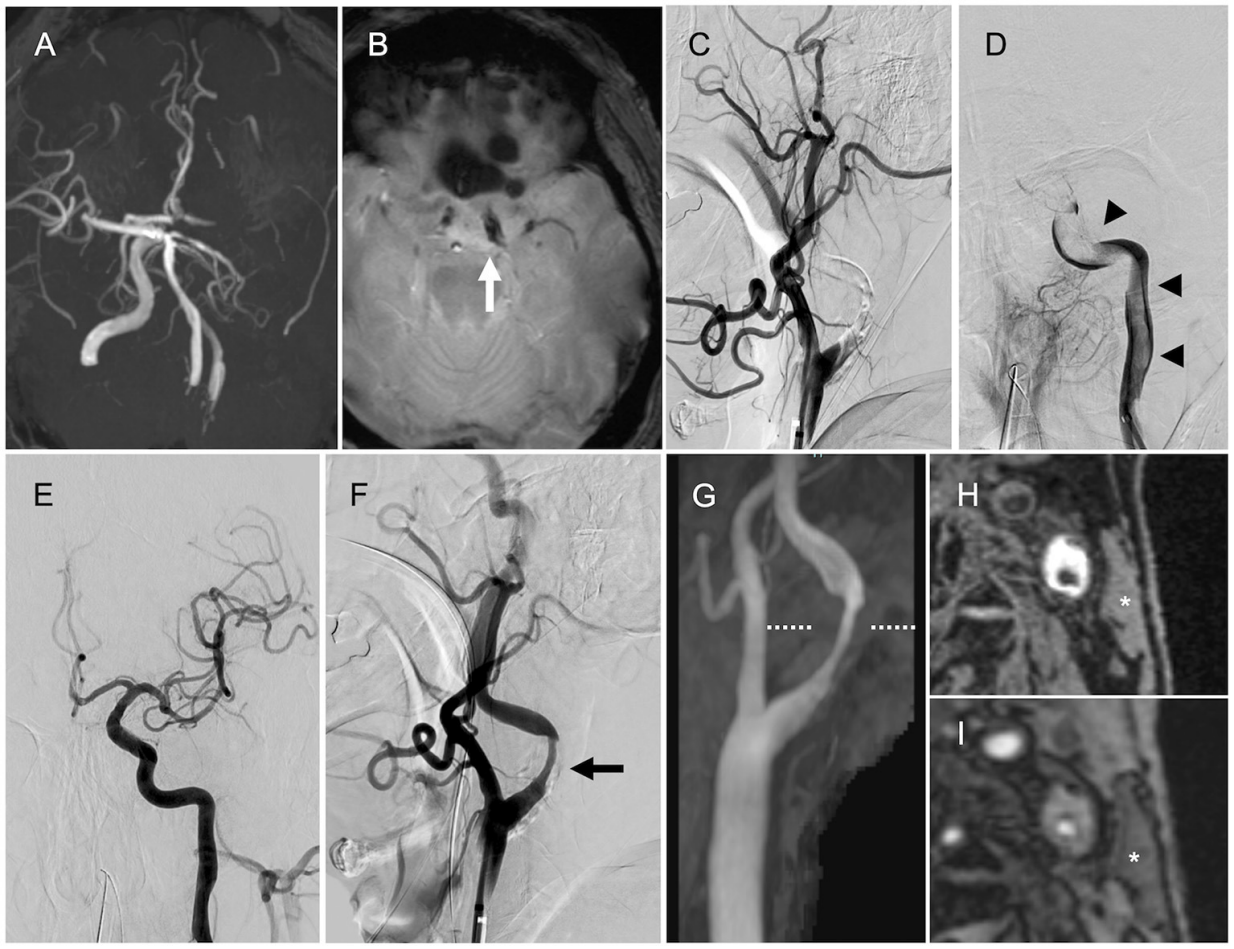
Magnetic resonance imaging (MRI) and angiography findings. **(A)** Magnetic resonance angiography (MRA) showing the left internal carotid artery (ICA) occlusion. **(B)** T2*-weighted MRI showing the susceptibility vessel sign at the top of the ICA (*arrow*). **(C)** Left internal carotid angiography showing the left proximal ICA occlusion (lateral view). **(D)** Angiography via the aspiration catheter in the cervical ICA showing a continuous filling defect (*arrowheads*) from the cervical ICA to the intracranial ICA (anteroposterior view). **(E)** Final angiography showing successful reperfusion (anteroposterior view). **(F)** Final angiography showing the left ICA stenosis (*arrow*, lateral view). **(G)** Neck MRA showing the left ICA stenosis. **(H,I)** Magnetization-prepared rapid gradient-echo image and maximum intensity projection images from time-of-flight MRA (level of the dotted line) showing high signal intensity in the plaque compared with the sternocleidomastoid muscle (*asterisk*).

A 69-year-old man with a history of hypertension presented with global aphasia and right hemiplegia of abrupt onset. He was transferred to our institute after 13 h after the last known well-time. His baseline National Institutes of Health Stroke Scale score was 24. Diffusion-weighted magnetic resonance imaging (MRI) revealed acute infarcts in the left parietal lobe. MR angiography revealed the left ICA occlusion (Figure 1A). T2^*^-weighted MRI performed as the routine protocol showed a susceptibility vessel sign at the top of the ICA (Figure 1B). Hence, MT was performed after obtaining informed consent for the procedure from the patient's family. Patient's consent was obtained for publication of this report.

### Intervention

The initial left common carotid angiography showed occlusion of the proximal ICA (Figure 1C). The aspiration catheter was navigated to the cervical ICA through the site of occluded segment of the proximal ICA. Angiography via the aspiration catheter revealed a large filling defect that extended from the cervical ICA to the intracranial ICA (Figure 1D). We deployed the stent retriever from the M1 segment of the middle cerebral artery (MCA) into the cavernous portion of the ICA. After the microcatheter was removed, the aspiration catheter was advanced to the intracranial ICA until the drip rate slowed. Then, we pulled the stent retriever and aspiration catheter as a unit into the balloon-guiding catheter. Successful reperfusion was achieved with a total of five passes by the above technique (four passes with Solitaire Platinum® 6 × 40 mm, Medtronic, Irvine, California, USA, and Penumbra ACE 60 aspiration catheter, Penumbra Inc., Alameda, CA, USA; one pass with Trevo® 4 × 20 mm, Stryker Neurovascular, Fremont, California, USA, and Penumbra ACE 60 aspiration catheter). Large red thrombi were retrieved with each pass. The final angiogram showed modified Thrombolysis in Cerebral Ischemia grade 2b reperfusion and stenosis in the proximal ICA (Figure 1E). The stenosis rate was 65% according to North American Symptomatic Carotid Endarterectomy Trial criteria (Figure 1F).

### Postoperative Examinations and Histopathological Findings

After the MT, we performed transthoracic and transesophageal echocardiography, along with electrocardiographic monitoring as the diagnostic workup of the embolic source, to determine if there was any other potential embolic source apart from the stenotic left ICA. However, no cardiac embolic sources were detected. Carotid artery ultrasonography showed severe stenosis with an echolucent plaque in the proximal ICA. Peak systolic flow velocity was 1.9 m/s. We have added neck MRI to evaluate the characteristics of the carotid plaque. Neck magnetization-prepared rapid gradient-echo image and maximum intensity projection images from time-of-flight MR angiography showed high signal intensity in the plaque compared with the sternocleidomastoid muscle (Figures 1G–I), indicating a vulnerable plaque. Although there were no embolic sources other than the carotid plaque, the possibility of cardiac source of embolism including covert atrial fibrillation could be completely excluded because of the large volume of retrieved thrombi atypical of LAA.

**Figure 2:**
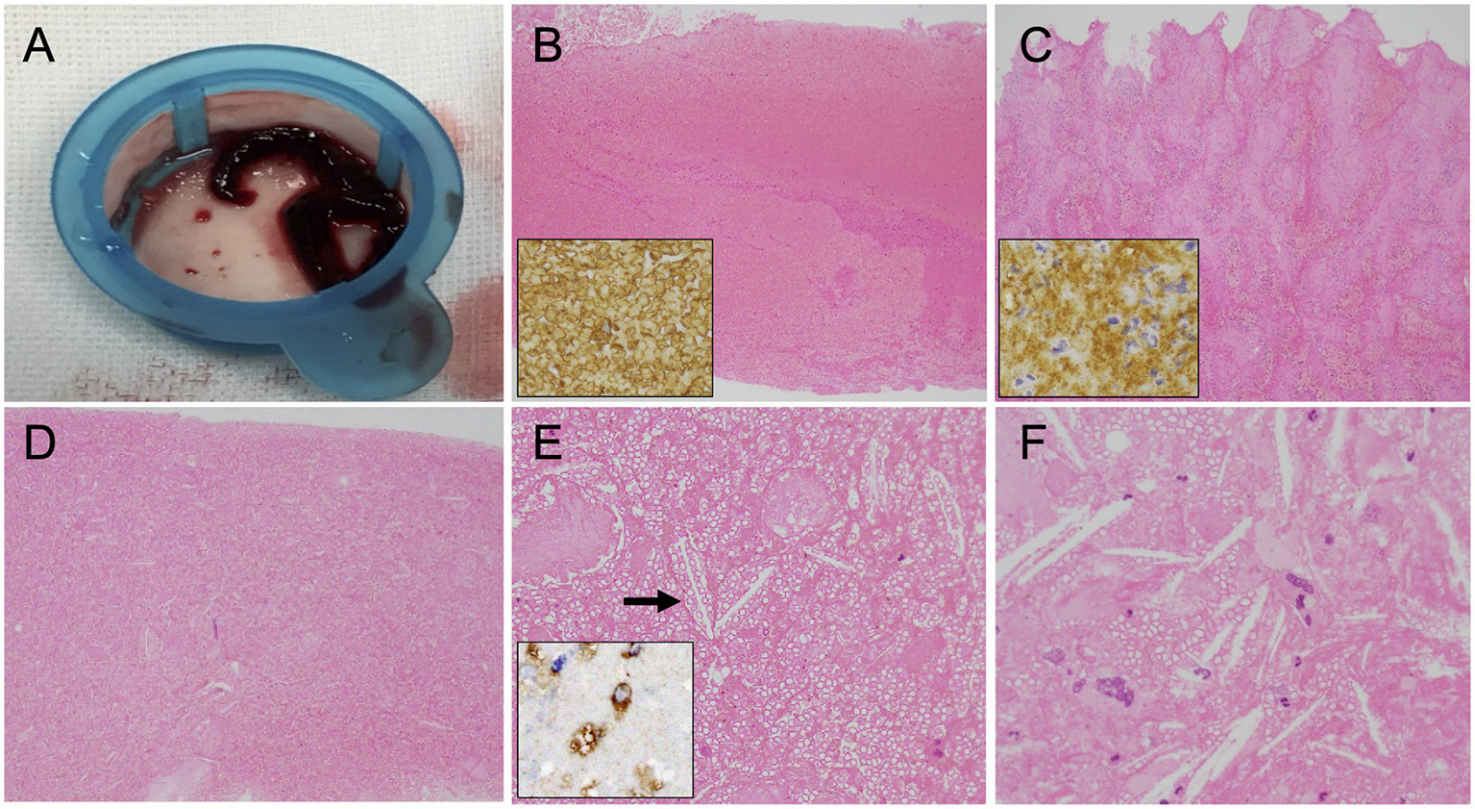
Macroscopic and microscopic findings of the retrieved thrombi. **(A)** Macroscopic appearance of the retrieved thrombi. **(B,C)** Histopathological sections showing the erythrocyte-rich thrombus that was immunopositive for anti-glycophorin A (insert) **(B)** and the platelet-rich thrombus that was immunopositive for anti-glycoprotein IIb/IIIa (insert) **(C)**. Original magnification ×40. **(D)** Many cholesterol clefts are distributed throughout this thrombus specimen. Original magnification ×40. **(E)** Cholesterol clefts (*arrow*) and foam cells in the erythrocyte-rich thrombus. Original magnification ×200. Insert immunohistochemistry with CD68 revealing the presence of macrophages. **(F)** Necrotic core with aggregation of cholesterol clefts and multinucleated giant cells in the platelet-rich thrombus. Original magnification ×400.

The specimens retrieved by MT were histopathologically analyzed. The obtained thrombi were fixed in phosphate-buffered formalin solution. Formalin-fixed specimens were embedded in paraffin, cut at 5 μm thickness, and stained with hematoxylin–eosin. Some retrieved thrombi specimens were in addition tested immunohistochemically to confirm the presence of erythrocytes, platelets, and macrophages. Images of the stained thrombi were acquired using a cellSens imaging software (Olympus Corporation, Tokyo, Japan) equipped with a light microscope (Nikon eclipse Ni, Nikon Corporation, Tokyo, Japan). Macroscopically, all retrieved thrombi were dark red in color (Figure 2A). Microscopic observation revealed that most of the thrombi were mainly composed of red blood cells (Figure 2B), although thrombi composed primarily of platelets were also observed (Figure 2C). The thrombus retrieved on the third pass was a red thrombus with diffuse cholesterol clefts (Figure 2D) and with foam cells scattered in the thrombus (Figure 2E). A necrotic core with aggregation of cholesterol clefts and multinucleated giant cells was also found in the platelet-rich thrombus (Figure 2F). Based on clinical and histopathological evaluations, his stroke etiology was determined as LAA.

Aspirin (100 mg/day) and clopidogrel (75 mg/day) were administered for secondary prevention of thromboembolism. His modified Rankin Scale score at 90 days after stroke onset was 1. Clopidogrel was discontinued at 3 months after treatment initiation. He had no recurrent ischemic stroke for 2 years after the index stroke.

## Discussion

We described here a patient who underwent MT for acute ICA occlusion due to LAA. Angiography during MT demonstrated a large thrombus extending from the cervical to the intracranial ICA. Histopathological examinations demonstrated that the retrieved thrombi had a variety of characteristics, including cholesterol clefts, foam cells, and a necrotic core. These characteristics of thrombi might be a histological signature of stroke due to LAA.

In percutaneous coronary intervention for acute coronary syndrome, plaque components including vessel wall fragments, a necrotic core, cholesterol crystals, and calcification were found in aspirated coronary materials in 44% of patients (3). In MT for acute ischemic stroke, on the other hand, there is only one report on the existence of the above components in retrieved thrombi (4). Atheromatous tissues were considered to be derived from atherosclerotic plaques or concomitant plaque components retrieved with vessel walls (4). The existence of atherosclerotic components was reportedly rare and less frequently observed in thrombi by recent thrombectomy devices (stent retriever or aspiration catheter) due to less damage to the arterial wall (4–6). Since there are no data on the association between atheromatous tissues in the thrombus and the detailed stroke etiology, its diagnostic role for embolic stroke remains unclear. In the present case, cholesterol clefts were widely distributed in the thrombi retrieved using recent thrombectomy devices, indicating that atheromas were involved in the *in situ* thrombus formation process on the atherosclerotic plaque. Those findings were consistent with the ipsilateral carotid vulnerable plaque. Initially, the possible cardiac source of embolism could not be completely ruled out because of the large thrombus volume. The existence and distribution of atheromas in thrombi reinforced the diagnosis of LAA. Especially in patients who have multiple candidates of embolic sources including atherosclerotic lesions, histopathological examination of retrieved thrombi might be useful to identify the embolic source.

A previous study reported that patients with LAA are likely to have smaller clot burden than those with cardioembolic stroke (6). However, the present case had a large quantity of thrombotic tissue and the character of the thrombi varied considerably, ranging from erythrocyte-rich to platelet-rich. In addition to the initial thrombus that causes vessel occlusion, some thrombi might form after vessel occlusion secondary to blood stasis. We speculate that, in our patient, atherosclerotic plaque rupture caused occlusion of the proximal ICA due to *in situ* thrombus formation, while intracranial ICA occlusion occurred because of artery-to-artery embolism. The large thrombus volume found in the occluded segment of the ICA might have formed secondary to the occlusion. Since massive thrombi can form even in LAA, the amount of thrombus might not be a definitive diagnostic feature when determining stroke etiology.

## Conclusion

We described here the histopathological report of thrombi retrieved by MT in case of LAA. In this case, we speculated that thrombi with atherosclerotic components developed at the site of the carotid plaque. Atherosclerotic components in retrieved thrombi might provide relevant information for determining stroke subtype. Further studies are warranted to clarify the utility of atheromatous components in the retrieved thrombi in the diagnosis of stroke etiology.

## Data Availability Statement

The raw data supporting the conclusions of this article will be made available by the authors, without undue reservation.

## Ethics Statement

Ethical review and approval was not required for the study on human participants in accordance with the local legislation and institutional requirements. Written informed consent for participation was not required for this study in accordance with the national legislation and the institutional requirements. Written informed consent was obtained from the individual(s) for the publication of any potentially identifiable images or data included in this article.

## Author Contributions

FE collected the data and wrote the manuscript. JK collected and analyzed the data and wrote the manuscript. KTa and KH collected the data and revised the manuscript. TY and MS collected the data. KTo supervised the manuscript. MK revised the manuscript. All authors contributed to the article and approved the final version of the manuscript.

## Conflict of Interest

The authors declare that the research was conducted in the absence of any commercial or financial relationships that could be construed as a potential conflict of interest.
